# Modeling radiation injury-induced cell death and countermeasure drug responses in a human Gut-on-a-Chip

**DOI:** 10.1038/s41419-018-0304-8

**Published:** 2018-02-14

**Authors:** Sasan Jalili-Firoozinezhad, Rachelle Prantil-Baun, Amanda Jiang, Ratnakar Potla, Tadanori Mammoto, James C. Weaver, Thomas C. Ferrante, Hyun Jung Kim, Joaquim M. S. Cabral, Oren Levy, Donald E. Ingber

**Affiliations:** 1 Wyss Institute for Biologically Inspired Engineering at Harvard University, Boston, MA 02115 USA; 2 Department of Bioengineering and iBB - Institute for Bioengineering and Biosciences, Instituto Superior Técnico, Universidade de Lisboa, Lisboa, Portugal; 3 Vascular Biology Program and Department of Surgery, Boston Children’s Hospital and Harvard Medical School, Boston, MA 02115 USA; 4 Department of Biomedical Engineering, The University of Texas at Austin, Austin, TX 78712 USA; 5 Harvard John A. Paulson School of Engineering and Applied Sciences, Cambridge, MA 02139 USA

## Abstract

Studies on human intestinal injury induced by acute exposure to γ-radiation commonly rely on use of animal models because culture systems do not faithfully mimic human intestinal physiology. Here we used a human Gut-on-a-Chip (Gut Chip) microfluidic device lined by human intestinal epithelial cells and vascular endothelial cells to model radiation injury and assess the efficacy of radiation countermeasure drugs in vitro. Exposure of the Gut Chip to γ-radiation resulted in increased generation of reactive oxygen species, cytotoxicity, apoptosis, and DNA fragmentation, as well as villus blunting, disruption of tight junctions, and compromise of intestinal barrier integrity. In contrast, pre-treatment with a potential prophylactic radiation countermeasure drug, dimethyloxaloylglycine (DMOG), significantly suppressed all of these injury responses. Thus, the human Gut Chip may serve as an in vitro platform for studying radiation-induced cell death and associate gastrointestinal acute syndrome, in addition to screening of novel radio-protective medical countermeasure drugs.

Exposure to ionizing γ-radiation, whether therapeutic or accidental, may result in acute radiation syndrome that is associated with gastrointestinal (GI) disturbances leading to massive shortening or “blunting” of intestinal villi, disruption of tight junctions, increased apoptosis within the microvascular endothelium, mucosal barrier breakdown, inflammation, abdominal pain, diarrhea, and vomiting, which can result in intestinal hemorrhage, sepsis, and death^1–4^. Development of medical countermeasures (MCMs) to protect against the devastating effects of radiation is therefore of tremendous importance. Animal models have been primarily used for GI radiation research because they can mimic some of the clinical manifestations of radiation poisoning (e.g., vomiting, diarrhea), however, these in vivo models often fail to effectively mimic cellular mechanisms of radiation toxicities or drug mechanisms of action displayed in humans^5,6^. Ethical issues related to animal testing also present a considerable hurdle, particularly when it relates to studies on primates^7^. As a result, the mechanisms underlying the radiation-induced GI syndrome remain unclear, and this represents a major challenge with regards to discovery of new MCMs^8,9^.

Understanding of radiation-induced intestinal injury could be greatly facilitated by the availability of experimental in vitro models that recapitulate human cell and tissue responses to radiation; unfortunately, this has not been possible using existing culture systems. In particular, the 3D villus architecture and differentiated barrier functions of the intestine are known to contribute greatly to intestinal tissue responses to radiation. It is likely for this reason that past efforts, for example, using Transwell culture systems lined by human Caco-2 intestinal epithelial cells that grow as a flat monolayer failed to model radiation injury^8,10,11^. Furthermore, past in vitro models used to study intestinal responses to radiation did not incorporate a human vascular endothelium in the vicinity of the intestinal epithelium to mimic capillary blood vessels, which are situated very close to epithelial cells in the gut mucosa^11^. This is important because while intestinal stem cells have always been assumed to be the major mediator of radiation damage involved in development of the GI syndrome^12,13^, recent studies suggest that apoptosis within the microvascular endothelium may be a key mediator of radiation damage that, in turn, leads to stem cell dysfunction^14–16^.

To model radiation-induced damage in vitro, we adapted a recently described human Gut-on-a-Chip (Gut Chip) microfluidic culture device that is lined by human intestinal epithelium interfaced with a human vascular endothelium, which spontaneously differentiates and forms three-dimensional intestinal villi when cultured in the presence of flow and cyclic peristalsis-like deformations^10,17,18^. Here we show that this microfluidic human Gut Chip can be used to analyze the effects of γ-radiation on villus morphology, barrier function, cell–cell junctions, cellular toxicity, apoptosis, reactive oxygen species (ROS) generation, and DNA fragmentation in vitro. We also demonstrate that it can be used as a tool to evaluate the radiation-protecting effects of a potential radiation countermeasure drug, the small-molecule prolylhydroxylase inhibitor dimethyloxalylglycine (DMOG), which has been reported to protect small intestine against radiation damage by stabilizing hypoxia-inducible factor 1α and 2α (HIF-1α and HIF-2α)^19^.

## Results

### Establishing a human gut radiation injury model in vitro

**Fig. 1:**
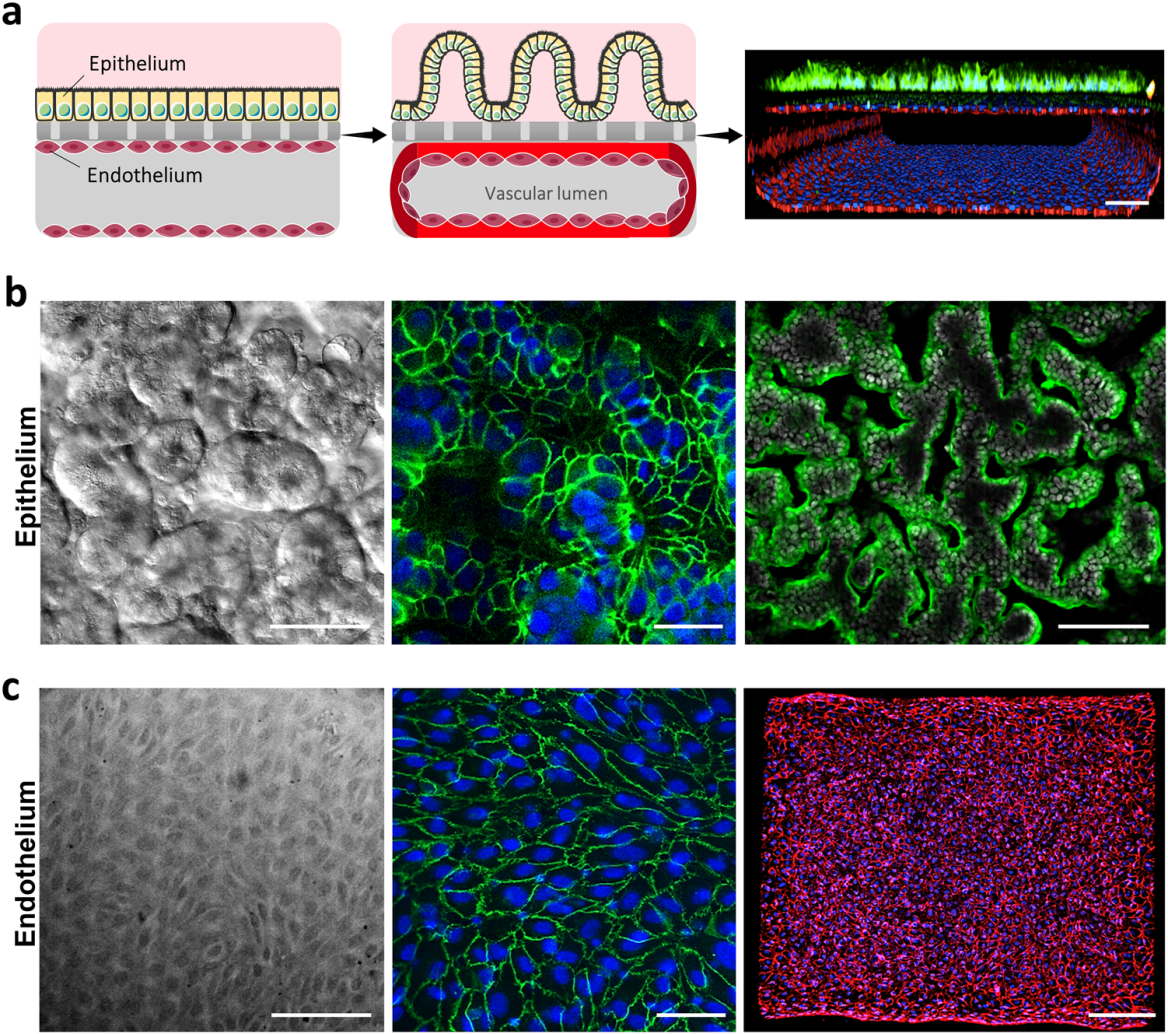
Human Gut Chip microfluidic culture device. **a** Schematic showing the positions of the human intestinal epithelium and endothelium when initially plated on opposite sides of the matrix-coated porous membrane within the two-channel microfluidic device (left), and how this progresses to form a villus epithelium in the top channel interfaced with a planar endothelium that forms a lumen in the bottom channel (middle). A representative immunofluorescence confocal micrograph visualizing a cross-section of the Gut Chip device with the villus intestinal epithelium stained for villin (green) to visualize the apical brush border, and the planar endothelium stained for VE-cadherin (red) to visualize adherens junctions, is shown at the right (bar, 100 μm). **b** Microscopic views showing the villus morphology of the human Caco-2 intestinal epithelium cultured for about 5 days in the Gut Chip with flow (30 μl h^−1^) and cyclic strain (10% at 0.15 Hz), when viewed from above by DIC imaging (left; bar, 50 μm) or by immunofluorescence staining for the tight junction protein, ZO-1 (green, middle; bar, 50 μm) and villin (green, right; bar, 100 μm). Blue and gray indicate DAPI-stained nuclei. **c** Microscopic views showing the planar morphology of the human endothelium cultured under identical conditions as in **b**, when viewed from above by phase contrast imaging (left; bar, 50 μm) or immunofluorescence staining for the endothelial cell junction-associated proteins PECAM-1 (green, middle; bar, 50 μm) and VE-cadherin (red, right; bar, 200 μm). Blue indicates DAPI-stained nuclei

The microfluidic human Gut Chip is a microfluidic culture device composed of a clear, flexible, poly-dimethylsiloxane (PDMS) polymer, which contains two parallel microchannels separated by a porous, flexible, extracellular matrix (ECM)-coated membrane lined by human Caco-2 intestinal epithelial cells on one side and human umbilical vein microvascular endothelial cells on the other (Fig. 1a, left). Medium is perfused through both channels (30 μl h^-1^; 0.02 dyne cm^−2^) and cyclic deformations (0.15 Hz; 10% strain) similar to those experienced by cells within the intestine during peristalsis^20^ are induced by applying cyclic suction through hollow side chambers (Supplementary Fig. S1a), as previously described^10,17,18^. When Caco-2 intestinal epithelial cells are cultured under these conditions, they undergo villus differentiation and express multiple features of human small intestine within 5–7 days when analyzed at the molecular, morphological, physiological, and transcriptomic levels^10,17,18^, even though the same cells in the same medium fail to undergo significant differentiation in Transwell cultures^10^. In contrast to past Gut Chip studies, we cultured endothelial cells on all four sides of the lower channel to engineer a hollow endothelium-lined vascular lumen in these devices (Fig. 1a, middle). Differential interference contrast (DIC) and immunofluorescence microscopic analysis confirmed that the Caco-2 cells formed a villus intestinal epithelium with polarized epithelial cells lined by ZO-1-containing tight junctions and an apical villin-containing brush border (Fig. 1b; Supplementary Fig. S1b). In the lower channel, endothelial cells grew to form a confluent cell monolayer joined by CD31-containing adherens junctions, which covered all four sides of the channel surrounding the hollow vascular lumen (Fig. 1a, right; Fig. 1c; Supplementary Fig. S1b).

**Fig. 2:**
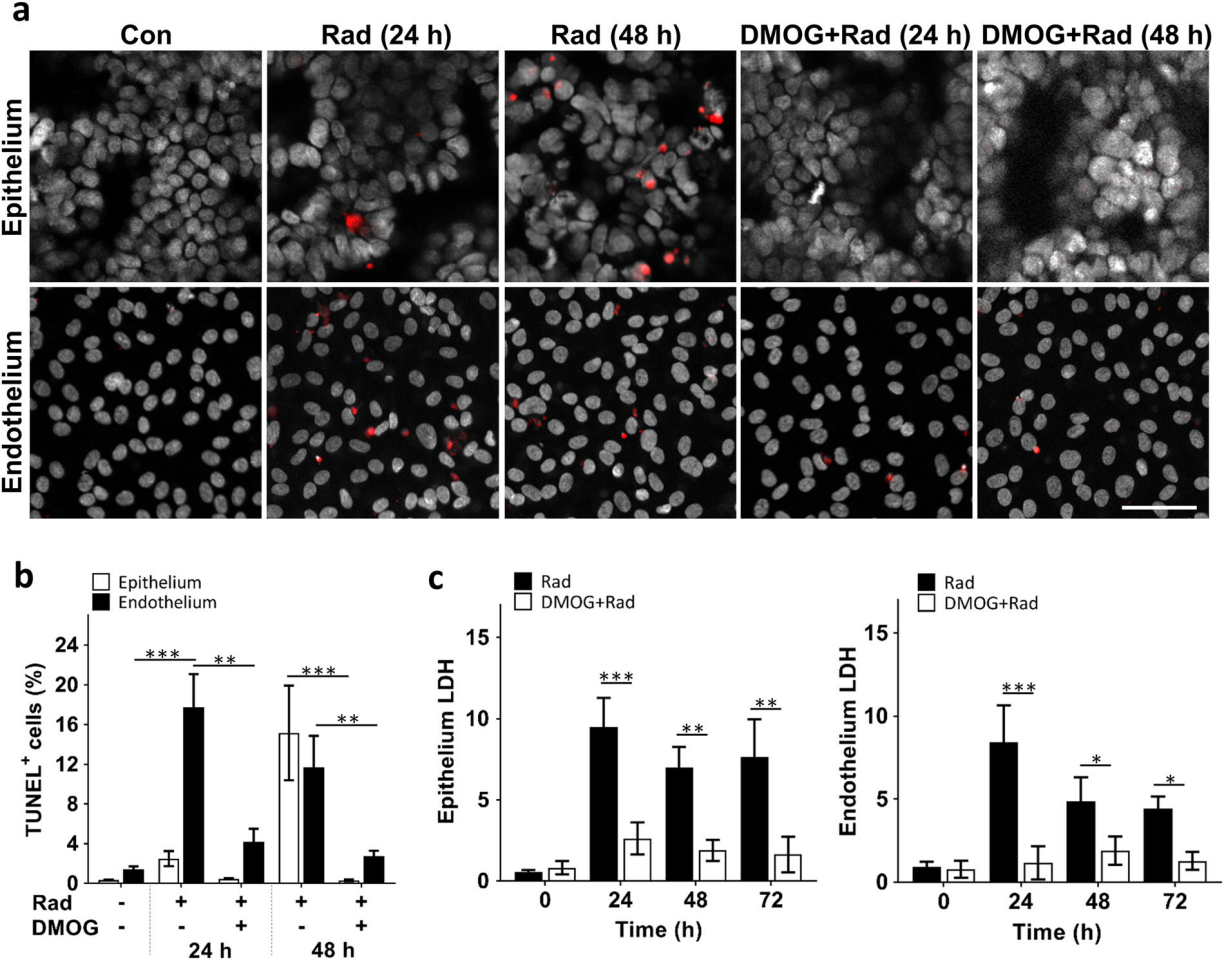
Radiation-induced apoptosis and cytotoxicity in intestinal epithelium and vascular endothelium, and radio-protective effects of DMOG. **a** Representative immunofluorescence micrographs of TUNEL (red) and DAPI (white) staining in epithelial and endothelial cells cultured on-chip in the absence (Con) or presence (Rad) of 8 Gy of γ-radiation (Rad), with or without DMOG treatment, 24 h and 48 h after radiation exposure (bar, 50 μm). **b** Graph showing the quantification of the percentage of epithelium and endothelium cells that expressed TUNEL staining (TUNEL^+^ cells) 24 h and 48 h after exposure to the conditions shown in **a** (*n* = 3; ^*^P < 0.05, ^**^P < 0.01, ^***^P < 0.001). **c** Graph showing radiation-induced cell death in the epithelium (left) and endothelium (right) in the absence (Rad) or presence of DMOG (DMOG + Rad), as assessed by quantifying LDH release from cells (data are presented as fold change in LDH levels relative to the non-irradiated control cells; *n* = 3; ^*^P < 0.05, ^**^P < 0.01, ^***^P < 0.001)

We then exposed the human gut chips to γ-radiation. Previous studies have shown that exposure to a level of ~4-8 gray (Gy) of γ-radiation can lead to GI syndrome and death in the absence of treatment in humans^13,21^. Rodents subjected to total body irradiation at a dose of 8 Gy also show progressive intestinal injury with increased number of apoptotic and mitotic cells, as well as shortening of the villi^22,23^. Exposure of the human gut chips to 8 Gy γ-radiation for 24 h similarly resulted in significant increases in apoptosis within both epithelium and endothelium compared to non-irradiated control chips, however, the number of apoptotic endothelial cells was about fivefold higher in the endothelium at this time (Fig. 2a,b). Interestingly, the epithelium appeared to undergo a distinct and slower mechanism of apoptosis induction as the apoptosis labeling index increased significantly 48 h after radiation exposure, whereas the endothelial apoptosis index was maximal at  day 1 (Fig. 2a,b). In contrast, both the epithelium and endothelium exhibited similar levels of cell membrane damage at 24 h, as measured by quantifying extracellular release of the intracellular enzyme, lactate dehydrogenase (LDH), and these levels remained high for at least 72 h after radiation exposure (Fig. 2c). Control studies also confirmed that direct exposure of the chip without cells did not induce cytotoxicity when cells were then cultured on these devices (Supplementary Fig. S2**)**, confirming that cell injury was not caused by radiation-induced release of toxins from the PDMS material.

**Fig. 3:**
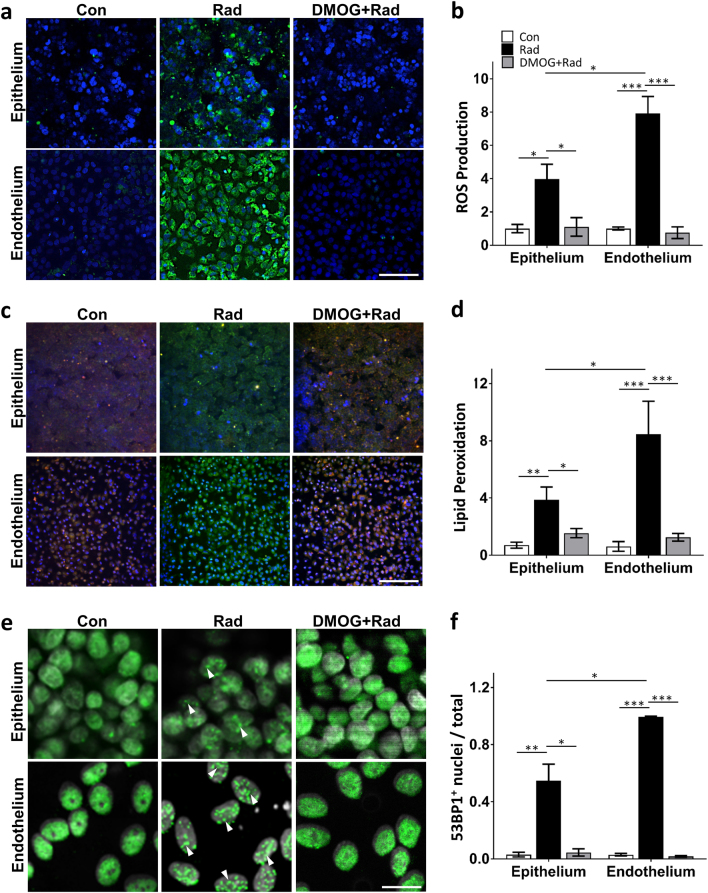
Radiation-induced changes in ROS, lipid peroxidation and DNA fragmentation in the presence or absence of DMOG treatment. **a** Radiation-induced changes in intracellular ROS in the intestinal epithelium (top) and endothelium (bottom) measured 30 min after exposure to γ-radiation (8 Gy) in the absence (Rad) or presence of DMOG (DMOG + Rad), or under control non-radiated conditions (Con), as visualized using the CellROX Green Reagent (green) (blue, DAPI-stained nuclei; bar, 50 μm). **b** Quantification of ROS production measured under the conditions described in **a**, expressed as fold change relative to non-irradiated control cells (*n* = 3; ^*^P < 0.05, ^***^P < 0.001). **c** Radiation-induced changes in lipid peroxidation in the intestinal epithelium (top) and endothelium (bottom) measured under the same conditions as shown in **a**, detected using an Image-iT® Lipid Peroxidation Kit in which lipid peroxidation results in a shift of fluorescence emission from 510 nm (red) to 590 nm (green) (blue indicated DAPI-stained nuclei; bar, 50 μm). **d** Quantification of lipid peroxidation measured under the conditions described in **c**. Data are presented as a shift the ratio of the fluorescence emission peak from 510 nm to 590 nm (*n* = 3; ^*^P < 0.05, ^**^P < 0.01, ^***^P < 0.001). **e** Effect of radiation on formation of DNA double-strand breaks, as detected by increased punctate staining of 53BP1-positive (53BP1^+^) nuclei (green); nuclei were counterstained with DAPI (white) (bar, 20 μm). White arrowheads indicate nuclei that display discrete punctate patterns of 53BP1^+^ staining. **f** Quantification of the ratio of nuclei that exhibited 53BP1^+^ punctate staining relative to total nuclei measured under the conditions described in **e** (*n* = 5; ^*^P < 0.05, ^**^P < 0.01, ^***^P < 0.001)

As ROS are critical mediators of radiation-induced damage that have been reported to play a pivotal role in intestinal epithelial injury as well as epithelial and endothelial apoptosis in patients^24–26^, we sought to determine if radiation induces ROS formation in the human Gut Chip. Quantification of ROS production using CellROX Green Reagent revealed that endothelial cells generated about twice the level of ROS per cell compared to the intestinal epithelial cells (approximately eightfold versus fourfold increases, respectively) (Fig. 3a,b). Lipids are one of the main targets attacked by ROS, and this results in lipid peroxidation and cell membrane damage^27–29^. Consistent with this observation and our ROS results, the level of lipid peroxidation after radiation was again almost two times higher in the endothelium compared to the epithelium (Fig. 3c,d).

Ionizing radiation is thought to produce many of its damaging effects on cells by producing clustered DNA double-strand breaks (DSB), promoting hyperphosphorylation of DSB-associated proteins, and inducing formation of discrete nuclear foci containing p53-binding protein 1 (53BP1), an important regulator of DSB signaling that is regulated by γ-radiation^30–32^. Similarly, we found that while 53BP1 was diffusely localized in the nuclei of control intestinal epithelial and endothelial cells in the Gut Chip, it relocated to punctate nuclear foci within both cell types when they were irradiated (Fig. 3e,f). Again, while more than 95% of endothelial cells exhibited discrete nuclear foci formation, the intestinal epithelial cells only exhibited about half this level of response. These findings appear to be consistent with past studies that suggested radiation-induced epithelial cell injury and DNA damage are mediated by endothelial apoptosis and ROS generation which are upstream from the epithelial cell injury responses^15,33–35^.

### Recapitulating intestinal organ-level radiation-induced injury

**Fig. 4:**
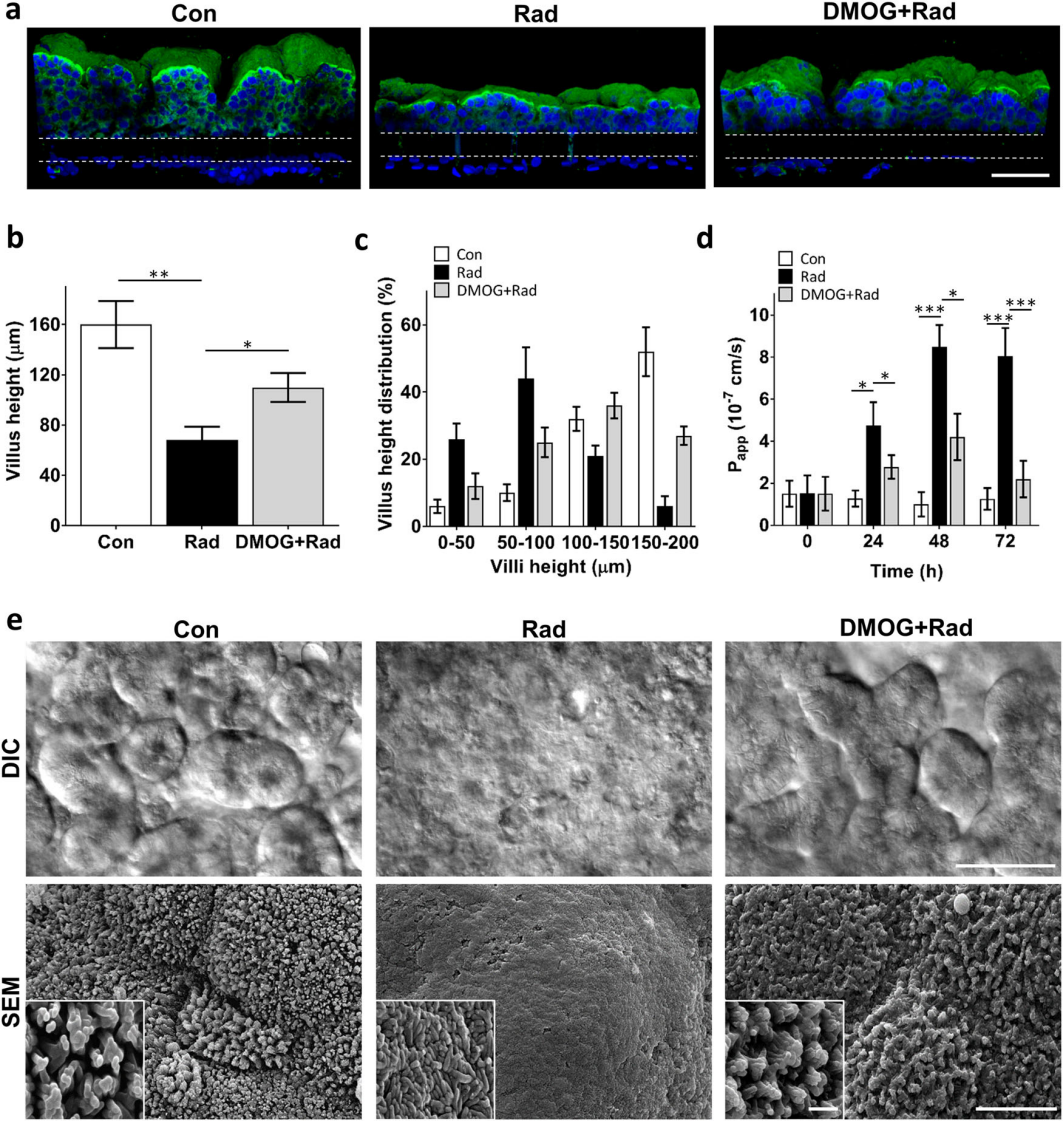
Morphological analysis of intestinal villus damage induced by radiation exposure. **a** Representative vertical cross-sectional, confocal, micrographic views through the intestinal epithelium-porous membrane-endothelium interface of the Gut Chip at 48 h before (Con) or after exposure to γ-radiation (8 Gy) in the absence (Rad) or presence of DMOG pre-treatment (DMOG + Rad), when immunostained for villin (green) and nuclei with DAPI (blue) (parallel white-dashed lines indicate upper and lower surfaces of the porous matrix-coated membrane; bar, 100 μm). **b** Quantification of intestinal injury evaluated by measuring changes in the height of the villi (*n* = 50) under control conditions (Con) versus after exposure to γ-radiation (8 Gy) in the absence (Rad) or presence of DMOG pre-treatment (DMOG + Rad) (^*^P < 0.05, ^**^P < 0.01). **c** Distribution of villus heights measured under the conditions described in **b**. **d** Changes in apparent paracellular permeability (*P*_app_) measured by quantitating cascade blue transport across the tissue-tissue interface within the Gut Chip microdevices before (Con) or after radiation in the absence (Rad) or presence of DMOG pre-treatment (DMOG + Rad) (*n* = 3; ^*^P < 0.05, ^***^P < 0.001). **e** DIC (top; bar, 50 μm) views of the intestinal villi and SEM micrographs (bottom; low magnification bar, 10 μm; high magnification inset bar, 1 μm) of the intestinal microvilli formed on-chip under control conditions (Con) or after radiation exposure without (Rad) or with DMOG pre-treatment (DMOG + Rad)

**Fig. 5:**
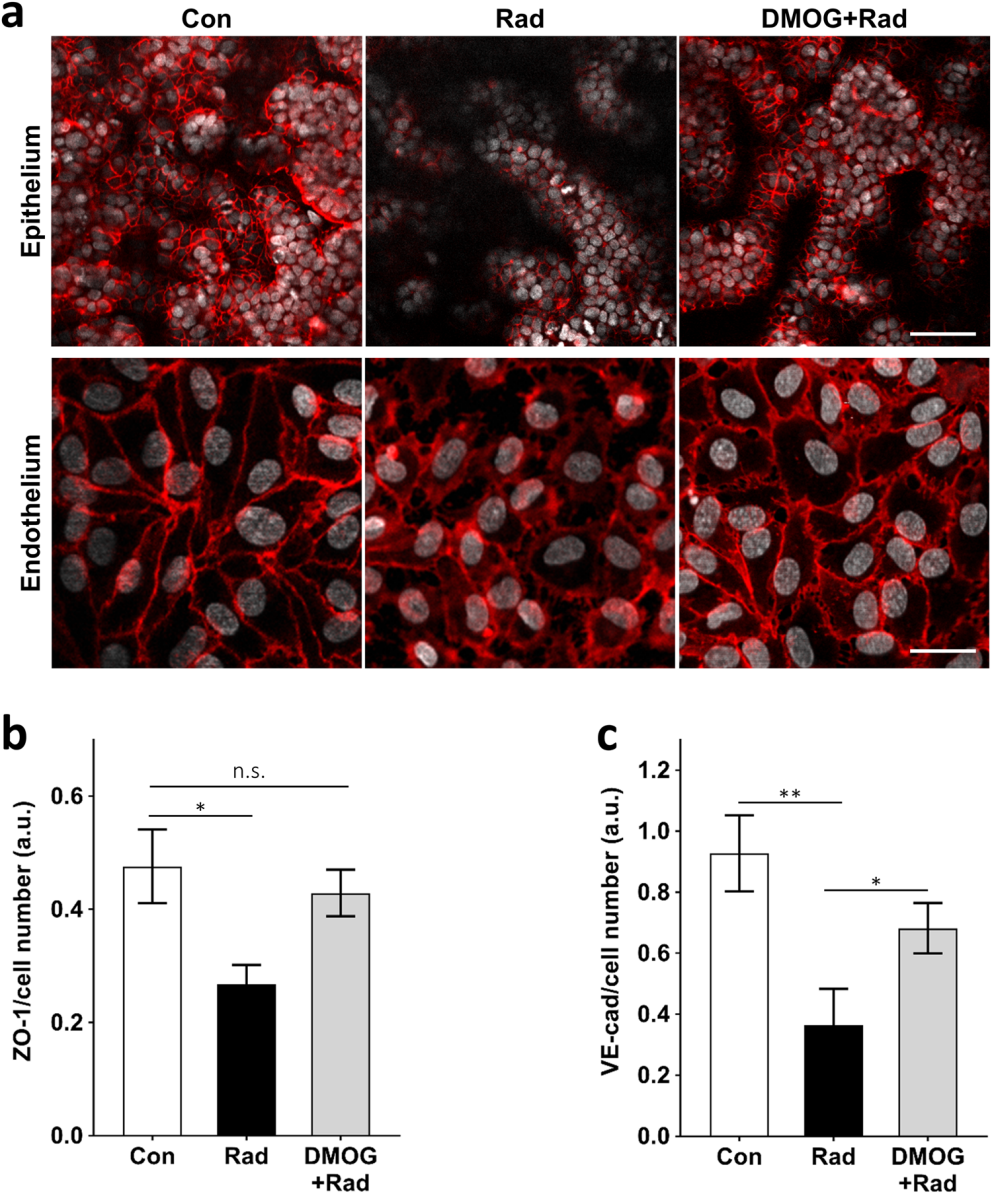
Radiation-induced loss of junctional continuity and mucosal damage and the effects of DMOG pre-treatment. **a** Confocal immunofluorescence micrographs showing horizontal views through the mid-section of villi (100 μm above the porous membrane) stained for epithelial tight junctions (ZO-1, red) at the top (bar, 100 μm), and through the middle of the endothelium stained for adherens junctions (VE-cadherin, red) at the bottom (bar, 20 μm), within non-irradiated Gut Chips (Con) versus Gut Chips exposed to 8 Gy γ-radiation for 48 h without (Rad) or with DMOG pre-treatment (DMOG + Rad). Quantification of fluorescence intensities (a.u.) of (**b**) ZO-1 and (**c**) VE-cadherin normalized per cell number (*n* = 3; ^*^P < 0.05, ^**^P < 0.01; n.s. not significant)

The small intestines of patients exposed to high levels of γ-radiation exhibit characteristic morphological changes including decreased villi height and irregular, shortened microvilli (“villus blunting”), as well as cytoplasmic vacuolization and detachment of epithelial and endothelial cells from their basement membrane^36–39^. Similarly, we found that exposure of the Gut Chips to 8 Gy resulted in loss of normal villus architecture and disruption of epithelial and endothelial integrity within 48 h. Computerized image analysis of cross-sectional immunofluorescence views of villin-stained epithelium (Fig. 4a) revealed a significant reduction in the average height of villi in irradiated versus control chips (68.2 ± 11.3 vs. 160.4 ± 19.6 μm, respectively) (Fig. 4b). Moreover, while the heights of the majority of the finger-like villi in control (non-irradiated) chips ranged between 100 and 200 μm, <35% of villus structures in the irradiated chip reached 100 μm or above (Fig. 4a,c). In addition, scanning electron microscopic (SEM) analyses showed that the apical surface of the irradiated epithelial cells had very short and irregular microvilli compared to control samples, making it difficult to identify cell boundaries in the irradiated epithelium (Fig. 4e). When we analyzed the expression of the cell–cell junction proteins, ZO-1 and VE-cadherin, in the irradiated epithelial and endothelial cells, respectively, we observed decreased expression of both molecules and a loss of junctional continuity in the cell layers (Fig. 5a-c). Radiation exposure also resulted in cell detachment and generation of cell-free gaps in the endothelial monolayer.

**Fig. 6:**
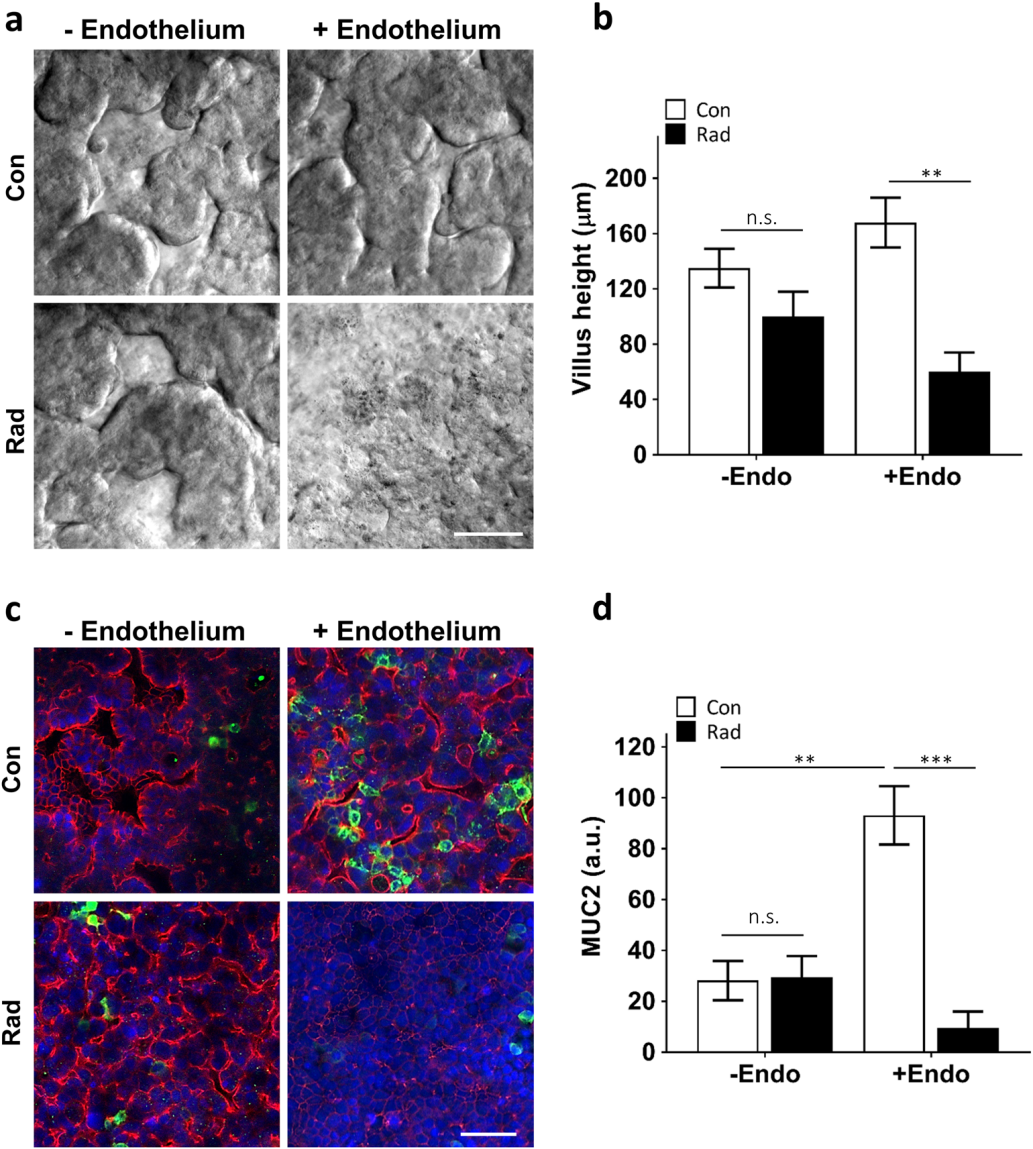
Vascular endothelium mediates the radiation damage. **a** DIC microscopic views showing the villus morphology of the human Caco-2 intestinal epithelium cultured with and without human vascular endothelium under control conditions (Con) or after radiation exposure (Rad) (bar, 50 μm). **b** Quantification of villus heights measured under the conditions described in **a** (*n* = 3; ^**^P < 0.01; n.s. not significant). **c** Representative immunofluorescence confocal micrographs of the Gut Chip device with the villus intestinal epithelium stained for epithelial tight junctions, ZO-1 (red) and intestinal mucin protein, MUC2 (green) (blue, DAPI-stained nuclei; bar, 50 μm). **d** Quantification of fluorescence intensities (a.u.) of MUC2 normalized per cell number under the conditions shown in **c** (*n* = 3; ^**^P < 0.01, ^***^P < 0.001; n.s. not significant)

Radiation injury of intestinal villi in humans is also accompanied by breakdown of the intestinal mucosal barrier, which can release bacteria and their toxins through the intestinal wall, leading to further abdominal complications^40–42^. When we evaluated the effect of radiation on intestinal barrier function and mucosal damage by measuring changes in the apparent permeability coefficient (*P*_app_), which is a measure of paracellular barrier function in the human Gut Chip, we observed a fourfold increase in *P*_app_ (4.9 × 10^−7^ vs. 1.2 × 10^−7^ cm s^−1^) at 24 h after exposure when significant apoptosis was detected in the endothelial cells, and *P*_app_ almost doubled again (8.7 × 10^−7^ cm s^−1^) by 48 h (Fig. 4d). This was also accompanied by significant loss of expression of the mucin protein, MUC2 (Fig. 6c,d), which is the most abundant mucin secreted by goblet cells that plays a pivotal role in organizing the intestinal mucus layers and barrier integrity at the epithelial apical surface^43,44^. Importantly, the suppression of MUC2 levels we detected on-chip is similar to what is observed in irradiated intestinal tissue in vivo^45^. Thus, the Gut Chip faithfully mimics many facets of the human intestinal injury response to radiation in vitro.

### Endothelial cells as mediators of radiation damage

To confirm whether vascular endothelium contributes to radiation-induced intestinal epithelium damage, we irradiated the Gut Chips in the absence or presence of endothelial cells and analyzed effects on radiation-induced epithelium damage at cell and tissue levels. Interestingly, whereas Gut Chips containing both epithelium and endothelium exhibited villus blunting in response to radiation exposure (Fig. 4), there was no significant decrease in average height of villi in irradiated chips lined by intestinal epithelium in the absence of endothelium (Fig. 6a,b). We also found that the presence of endothelial cells enhanced barrier function (reduced permeability by ~10-fold; Supplementary Fig. S3c) and increased mucus secretion (approximately fourfold; Fig. 6c,d) by the epithelium compared to chips lacking endothelium, and this was accompanied by suppressed or delayed responses to radiation damage. For example, ROS generation in irradiated epithelium was significantly lower when endothelium was not present (Supplementary Fig. S3a,b). Moreover, in the absence of endothelium, no changes in paracellular barrier function were observed even as late as 24 h and 48 h after radiation (Supplementary Fig. S3c). Thus, based on our observations, the endothelium appears to be the principal target of intestinal radiation injury, which is consistent with recent in vivo findings^46^.

### Radio-protective effects of a potential radiation countermeasure drug

Pharmacologic strategies for preventing or treating radiation-induced intestinal injury are currently very limited; however, a recent study showed that the prolylhydroxylase inhibitor, DMOG, partially prevented murine intestinal wall dysfunction when administered before abdominal irradiation^47^. DMOG elevates levels of HIF-1α and HIF-2α, which are known to promote cell survival under conditions of stress, such as ionizing radiation^19,48^. Similar to the in vivo finding, we found that DMOG pre-treatment prevented cell detachment (Fig. 5a-c), suppressed the increase in intestinal permeability (Fig. 4d), increased MUC2 expression (Supplementary Fig. 5a,b), and reduced villus injury (Fig. 4e), as evidenced by enhanced expression of ZO-1 (Fig. 5a) and increased villus height (Fig. 4a,b) in irradiated Gut Chips. In addition, we discovered that pre-treatment of cells with DMOG significantly reduced the number of cells undergoing apoptosis in both the epithelium and endothelium (Fig. 2b,c), in addition to inhibiting both ROS generation (Fig. 3a,b) and lipid peroxidation (Fig. 3c,d) in these cells. As expected this was associated with increased expression of HIF-1α and HIF-2α (Supplementary Fig. S4a-d), which is known to prevent ROS generation^49^ as well as inhibit apoptosis^50,51^. Interestingly, we also found that DMOG prevents DNA fragmentation (Fig. 3e,f), which has never been described previously. Thus, again, the human Gut Chip mimicked the protective effects of this potential radiation countermeasure drug that were previously observed in animal models of intestinal inflammation^51–53^.

## Discussion

In the present study, we leveraged a mechanically active, microfluidic Gut Chip device to model human intestinal radiation injury in vitro. The human Gut Chip recapitulates clinically relevant acute radiation injuries at both cellular and tissue–organ levels. Radiation induces loss of junctional continuity, compromises intestinal barrier function, and inhibits mucus production, in addition to promoting villus blunting and distortion of microvilli. Our data show that the endothelium appears to be more sensitive to radiation injury in terms of ROS generation, lipid peroxidation and DNA fragmentation, which first triggers rapid endothelial apoptosis that then appears to trigger subsequent epithelial cell injury responses, as previously described to in vivo^14,33^. Moreover, pre-treatment with a potential MCM drug, DMOG, significantly reduced radiation toxicity in epithelium and endothelium in this in vitro model, again mimicking responses that have been previously observed in vivo^47^.

Because of the urgent need for predictive intestinal radiation models, previous studies have explored the use of other types of in vitro cultures, including organoids and Transwell co-culture systems^11,54^. However, they were found to be limited in their ability to replicate normal human intestinal 3D architecture and function, and thus, they could not recapitulate important features of radiation responses, such as villus blunting, which have been observed in human patients. Our results suggest that these past models were ineffective because they failed to recreate the physiologically relevant physical microenvironment of the intestine, including fluid flow and peristalsis-like mechanical deformations, which are present in the human Gut Chip. Moreover, unlike our experimental system, past in vitro models did not contain human endothelial cells that are known to serve as key contributors to radiation-induced intestinal dysfunction^15,16^. For example, using the vascularized human Gut Chip, we found that the endothelium responds faster to ionizing radiation compared to the epithelium, with endothelial cells exhibiting an increase in apoptosis within 24 h after exposure, while epithelial cell death increased over the following day. This is consistent with the past finding that while exposure of mice to γ-radiation results in loss of intestinal villi beginning approximately 24–36 h after exposure, little apoptotic activity was observed in the intestinal epithelium during the first day^12,55^. Moreover, when Gut Chips were lined by epithelium in the absence of endothelium, we did not detect radiation-induced villus blunting, loss of barrier function, or decreased mucus secretion, confirming that endothelial cells mediate the intestinal radiation injuries as suggested by previous in vivo studies^15,16^. In addition, we found out that radiation-induced ROS generation by intestinal epithelium cultured on-chip alone was significantly lower than when it was co-cultured with endothelium. These data suggest that enhanced ROS generation by the endothelium may play a pivotal role in regulating intestinal radiation damage, and hence that it could serve as a target for therapeutics that mitigate radiation toxicity, as suggested by recent in vivo reports^25,46^. Although radiation damage is often coordinated by the intracellular actions of ROS^56^, further investigation of other potential radiation-mediators, such as inflammatory cytokines, are required to fully understand the cross-talk between epithelial and endothelial cells in response to radiation exposure.

In summary, the results we obtained are fully consistent with past studies which revealed that the microvascular endothelium is the initial target of radiation damage, and that endothelial apoptosis is upstream of damage to the intestinal epithelium^33,35,57^. Other studies have also demonstrated that radiation-induced epithelial cell injury and DNA damage are mediated via endothelial apoptosis and ROS generation^15,33–35^, which trigger cytoskeletal changes and structural alterations within the endothelial cell monolayer^58^.

The changes in endothelial cell and tissue architecture that we observed are consistent with the immediate morphological hallmarks of radiation that have been reported to occur in the vascular compartment in patients at clinically relevant doses^59,60^. In addition, the radiation-induced changes in the epithelium we observed in the Gut Chip, ranging from villus blunting to distortion of the tips of the villi to their complete villus, are consistent with clinical findings in patients and animals exposed to ionizing radiation^37,61^. A similar level of damage to apical microvilli at the apical epithelial cell surface also has been reported in both human and mouse irradiated tissues^36,62^.

Importantly, we demonstrated that the Gut Chip radiation model can be used to evaluate therapeutic response of potential radiation countermeasure drugs. As a proof-of-concept, we showed that DMOG, which is known to protect murine intestine against radiation damage by stabilizing HIF levels^63^, has similar effects in the human Gut Chip. Interestingly, the radio-protective effects of this drug on human cells and tissues have not been previously reported, and thus, our findings provide hope that this potential therapeutic approach could be useful in humans. In addition to replicating effects of DMOG previously observed at tissue and organ-level in vivo^47^, our results also unveiled some novel features of DMOG action at the cell level, including its ability to prevent DNA fragmentation, which also has not been previously described. In the future, our intestinal radiation model could be harnessed to study the safety and efficacy of other radioprotectants that contain a free radical-scavenging group, such as amifostine, which has been reported to reduce DSB accumulation^46,64^.

We used the established human Caco-2 intestinal epithelial cell line in the present study because it reproducibly undergoes intestinal differentiation and exhibits villi formation as well as transcriptional signatures reminiscent of the ileum when cultured within the mechanically active Gut Chip model^10,17,18^. However, two important caveats require consideration when interpreting the results of this study with respect to the effects of radiation on intestinal toxicity. The first is that the Caco-2 cell line was originally isolated from a colon tumor, and thus, while it can recapitulate many features of the human ileum when analyzed at the transcriptional level^18^, it does not fully express the phenotype of a normal human intestine. These tumor-derived Caco-2 intestinal epithelial cells also might differ in their sensitivity to radiation as compared to normal epithelial and endothelial cells, so further studies are required to fully elucidate the cross-talk between the two tissue layers in response to radiation. Specifically, this approach could be further strengthened by integrating primary human intestinal epithelial cells which might be more radio-sensitive into the model in the future (e.g., by isolating cells from clinical biopsy samples), and this could enable one to evaluate patient-specific responses to radiation as well. Second, while we were capable of replicating multiple clinical responses of intestinal epithelium and endothelium to radiation, other cells, including immune cells and commensal microbiome that are present within the intestinal microenvironment are believed to play a pivotal role in small intestine radiation toxicity in vivo^65,66^. Thus, it will be interesting to explore how the presence of these other cell types in the model might influence intestinal epithelial and endothelial responses to radiation in the future.

When animals or humans are exposed to total body radiation doses that lead to severe enterocyte depletion, lethality is mainly due to the combined effect of endothelial injury, GI damage, and bone marrow failure^7^. Therefore, past studies focused on preventing GI damage that utilized in vivo total body radiation models may therefore have had limited clinical relevance^46^. In contrast, the human Gut Chip radiation model offers the unique capability to analyze the direct effects of radiation toxicity on cells and tissues within a single organ, in the absence of contributions from other organs and systemic factors. However, different vascularized human Organ Chips can be linked by their common endothelium-lined vascular channels to create an effective “human Body-on-Chips”, which could then be used to explore how partial or whole body exposure to radiation modifies the response of intestinal tissues contained within a linked Gut Chip. Using this organ-level synthetic biology approach, it should be possible to identify how individual molecular, cellular, and physical factors contribute individually and in combination to radiation toxicity. In addition to enabling study of the underlying mechanisms involved in radiation-induced GI syndrome, the human Gut Chip offers a potentially powerful tool for discovery and screening of new and more effective countermeasure drugs in the future.

## Materials and methods

### Human Gut-on-a-Chip microdevice

The microfluidic Gut-on-a-Chip devices were fabricated from PDMS (SYLGARD® 184 silicone elastomer kit) by soft lithography technique as previously reported^17^. The intestinal epithelium and vascular endothelium channels shared same dimensions (1000 μm wide  × 14 mm long × 200 μm high) and separated by a porous (10 μm diameter pores with 25 μm spacing) PDMS membrane (50 μm high). Prior to cell seeding, both microchannels were activated using oxygen plasma (Diener ATTO; pressure, power, and plasma time were 0.5 mbar, 30 W, and 2 min, respectively), functionalized with (3-aminopropyl)trimethoxysilane (Sigma, 281778), washed by flowing absolute ethanol (Sigma, E7023) and dried in a 80 ^o^C oven. Microchannels were then coated with type I collagen (30 μg ml^−1^; Gibco, A10483-01) and Matrigel (100 μg ml^−1^; BD Biosciences, 356237) in the serum-free Dulbecco’s Modified Eagle Medium (DMEM; Gibco, 10564011) for 1 h at 37 ^o^C. To form vascular lumen, human endothelial cells (Human Umbilical Vein Endothelial Cells (HUVECs); Lonza, C2519A (pooled donor)) cultured in endothelial growth medium (DMEM/F12 (Gibco, 11039-021) plus EGM-2 BulletKit containing 100 U l^−1^ penicillin/100 mg ml^−1^ streptomycin (Pen/Strep; Gibco, 15140-122), hydrocortisone, hFGF-B, VEGF, R^3^-IGF-1, ascorbic Acid, hEGF, GA-1000, heparin, and 2% FBS; Lonza, CC-4176) were first introduced to the lower microchannel and incubated for 1 h to attach on bottom side of the channel. Afterwards, HUVECs were seeded on the opposite side of the porous membrane in the lower channel by immediate flipping the entire chip upside down and placing it in an incubator for 1 h. The device was flipped over again and human intestinal epithelial cells (Caco-2 BBE human colorectal carcinoma cell, Harvard Digestive Disease Center) grown in DMEM (Gibco, 10564011) containing Pen/Strep 20% Fetal Bovine Serum (FBS; Gibco, 10082-147) were seeded (1.5 × 10^5^ cells cm^−2^) into the top microchannel of the device and being allowed to adhere statically for 1 h. Gut chip was then cultured statically in incubator for an overnight to form monolayer. A day after seeding, epithelial medium (DMEM/20%FBS/antibiotics) and endothelial medium with reduced FBS (0.5%) were perfused at 60 μl h^−1^ through top and bottom channels, respectively. To mimic peristalsis-like motions, cyclic stretching (10% strain; 0.15 Hz) was applied through vacuum chambers via a vacuum controller 2 days after seeding. Five days after seeding, villus intestinal epithelium spontaneously appeared in top channel and endothelial vascular lumen formed in bottom channel. Endothelium-free chips were similarly prepared using the above procedure, except that after coating the chips with ECM, they were seeded with human intestinal epithelial cells (Caco-2) in top channel without adding endothelial cells to the bottom channel.

### Chip radiation

Gut-on-a-Chip microdevices containing villus intestinal epithelium (with or without endothelium) were removed from the syringe pump, immediately transferred to irradiation facility and exposed to a single 8 Gy dose of γ-irradiation (Cs-137; Gammacell 40 Exactor) at 0.98 Gy min^−1^. Temperature of irradiation chamber was kept at 37 °C through the procedure and no temperature fluctuations were observed. To treat the control chips similarly, they were brought to irradiation facility and back without being exposed to irradiation. To validate the prophylactic effect of dimethyloxalylglycine (DMOG) in our Gut-on-a-Chip devices, DMOG (Sigma, D3695) reconstituted in UltraPure DNase/RNase-free distilled water (Gibco, 10977015) and added to epithelium and endothelium channels at 1 mM an overnight before irradiation procedure. Control chips (vehicle) received only distilled water.

### Morphological analyses

Morphological analyses were performed using at least three independent gut chip samples at each interval, where images of villi were taken at more than 10 different locations. The intestinal villus morphology was evaluated using differential interface contrast (DIC) microscopy (Zeiss Axio Observer Z1 2, AXIO2). The villus microarchitecture was also studied using immunofloresencece microscopy with a laser scanning confocal microscopes (Leica SP5 X MP DMI-6000 and Zeiss TIRF/LSM 710) inked to a 405-nm diode laser, a white light laser (489–670 nm), or an argon laser (488 nm and 496 nm) and coupled to a photo-multiplier tube or HyD detectors. Acquired images were analyzed using IMARIS (MARIS 7.6 F1 workstation; Bitplane Scientific Software) and ImageJ. High-resolution horizontal or vertical cross-sectional images were obtained using deconvolution (Huygens) followed by a 2D projection process.

For SEM analysis, gut chips were designed in a way that top channel was not irreversibly bonded to the membrane, which permitted the device to be dismantled manually without disturbing the cultured cells. Cells were fixed with paraformaldehyde (PFA, 4%; Electron Microscopy Sciences, 157–4) and glutaraldehyde (2.5%; Sigma, G7776) and incubated in osmium tetroxide (0.5%; Electron Microscopy Sciences, 19152) before serial dehydration in ethanol. Samples were then dried using hexamethyldisilazane (Electron Microscopy Sciences, 999-97-3) and imaged with a field emission SEM (Tescan Mira GMU, Czech Republic).

### Paracellular permeability measurements

To measure intestinal permeability, 50 μg ml^−1^ of cascade blue (5.9 kDa; ThermoFisher, C687) were introduced to the top channel of the chips at 60 μl h^−1^ and fluorescence intensity of top and bottom channel effluents at excitation/emission wavelengths of 390 nm/420 nm were measured using a multi-mode plate reader (BioTekNEO) at different intervals. Apical-to-basolateral flux of the paracellular marker was calculated according to the following equation: *P*_app_ = (d*Q*/d*t*)/*A*×d*C*, where *P*_app_ (cm s^−1^) denotes the apparent permeability coefficient, d*Q*/d*t* (g s^−1^) is the molecular flux, *A* (cm^2^) is the total area of diffusion, and d*C* (mg ml^−1^) is the average gradient.

### Detection of reactive oxygen species and lipid peroxidation

Immediately after irradiation procedure, cellular ROS, and LPO were detected using CellROX Green (Life Technologies, C10444) and Image-iT Lipid Peroxidation Kit (Life Technologies, C10445), respectively, according to the manufacturer’s protocol. The signal intensity of intracellular levels of ROS was measured at excitation/emission wavelengths of 485 nm/520 nm (green), and ratios of emission peak from the 590 nm (red) to 510 nm (green) were used to quantify lipid peroxidation in chips.

### Cellular toxicity and apoptosis

*LDH activity assay:* CytoTox 96 non-radioactive cytotoxicity assay (LDH; Promega, G1780) was used according to the manufacturer’s instructions to measure epithelium and endothelium death rate at different intervals after irradiation procedure and DMOG treatments. In brief, effluents were collected from top and bottom channels, mixed with LDH substrate reagent and incubated for 30 min. The enzymatic reaction was terminated using stop solution (containing acetic acid) and the absorbance at 492 nm was recorded using a multi-mode plate reader (BioTekNEO). The LDH activity was assessed using quadruplicate of each group, calculated after subtracting the background absorbance values and reported as a fold change of the total LDH values of control group.

*TUNEL assay:* Late apoptosis was evaluated by the terminal deoxynucleotidyl transferase-mediated dUTP-biotin nick-end labeling (TUNEL) immunostaining using Click-iT TUNEL Alexa Fluor Assay Kit (Invitrogen, C10247) according to the manufacturer’s protocol. Chips were co-stained with DAPI (Invitrogen, D1306) as the nuclear DNA marker and the apoptotic cells were counted from 20 different fields (five fields each from four replicates) to get an average number of TUNEL-positive cells per field.

### Immunofluorescence microscopy

For immunofluorescence staining, cells in both channels were first gently washed with PBS, then fixed with PFA (4%; Electron Microscopy Sciences, 157–4) for 20 min and subsequently washed with additional PBS. Cells were then permeabilized with 0.25% Triton X-100 (0.25%; Sigma, T8787) for 20 min and incubated with blocking buffer containing 1% BSA (Sigma, A4503) and 10% donkey serum (Sigma, D9663) for 30 min at room temperature. Cells were then incubated with antibodies directed against ZO-1 (Life Technologies, 33-9100, dilution 1:200), PECAM-1/CD31 (eBioscience, BMS137, dilution 1:100), VE-cadherin/CD144 (BD Biosciences, 555661, dilution 1:200), Villin (Life Technologies, PA5-29078, dilution 1:100), 53BP1 (Abcam, ab36823, dilution 1:100), MUC2 (Santa Cruz Biotechnology, sc-15334, dilution 1:100), or HIF-1α (Abcam, ab16066, dilution 1:100) overnight at 4 ^o^C, followed by 6 × 5 min PBS washes. Secondary antibodies (Life Technologies) were then introduced in the channels for 1 h at room temperature and washed three times with PBS. Cells were co-stained with DAPI (Invitrogen, D1306). Microscopy was performed with a laser scanning confocal microscopy (Leica SP5 X MP DMI-6000 or Zeiss TIRF/LSM 710). Quantification of the immunofluorescence images was performed using ImageJ software based on the mean fluorescence intensity on a per cell basis.

### Western blot

To perform Western blot analysis, cell lysates were obtained using RIPA (Alfa Aesar, J63306) buffer supplemented with protease inhibitor cocktail (Roche). Followed by SDS-PAGE fractionation, samples were transferred to a nitrocellulose membrane according to the manufacturer’s protocol (BioRad). Westerns were run using precast gradient gels (4–15%, Biorad), with the same amount of protein load in each lane of an individual gel, and the relevant protein ranges were cut out and blotted with the individual antibodies. After blocking the membranes with 5% non-fat milk in TBST (50 mM Tris-HCl, 150 mM NaCl, 0.1% Tween-20), samples were incubated with antibodies against HIF-1α (R&D Systems, MAB1536, dilution 1:500), HIF-2α (Abcam, ab73895, dilution 1:500) and GAPDH (Santa Cruz Biotechnology, sc-32233, dilution 1:500). A horseradish-peroxidase-conjugated goat anti-rabbit or -mouse antibody was then added, and the membranes were developed with the ECL Plus system (GE Healthcare) according to the manufacturer’s protocol.

### Statistical analysis

All experiments were carried out at *n* = 3–6 (see figure captions), and results and error bars in this article are presented as mean ± standard error of the mean (s.e.m). Data analysis was performed with a one-way analysis of variance with Tukey HSD post hoc tests, using Graphpad Prism software. Statistical analysis between two conditions was performed by an unpaired Student’s *t* test. *P* values of less than 0.05 were considered to be statistically significant (*P < 0.05, **P < 0.01, ***P < 0.001).

## Electronic supplementary material

Figure S1

Figure S2

Figure S3

Figure S4

Figure S5

Supplementary Legend
