# Supplementary figures and images for: Modeling radiation injury-induced cell death and countermeasure drug responses in a human Gut-on-a-Chip
Source: Cell Death Dis. 2018 Feb 14;9(2):223. doi: 10.1038/s41419-018-0304-8 (PMC5833800; doi:10.1038/s41419-018-0304-8)

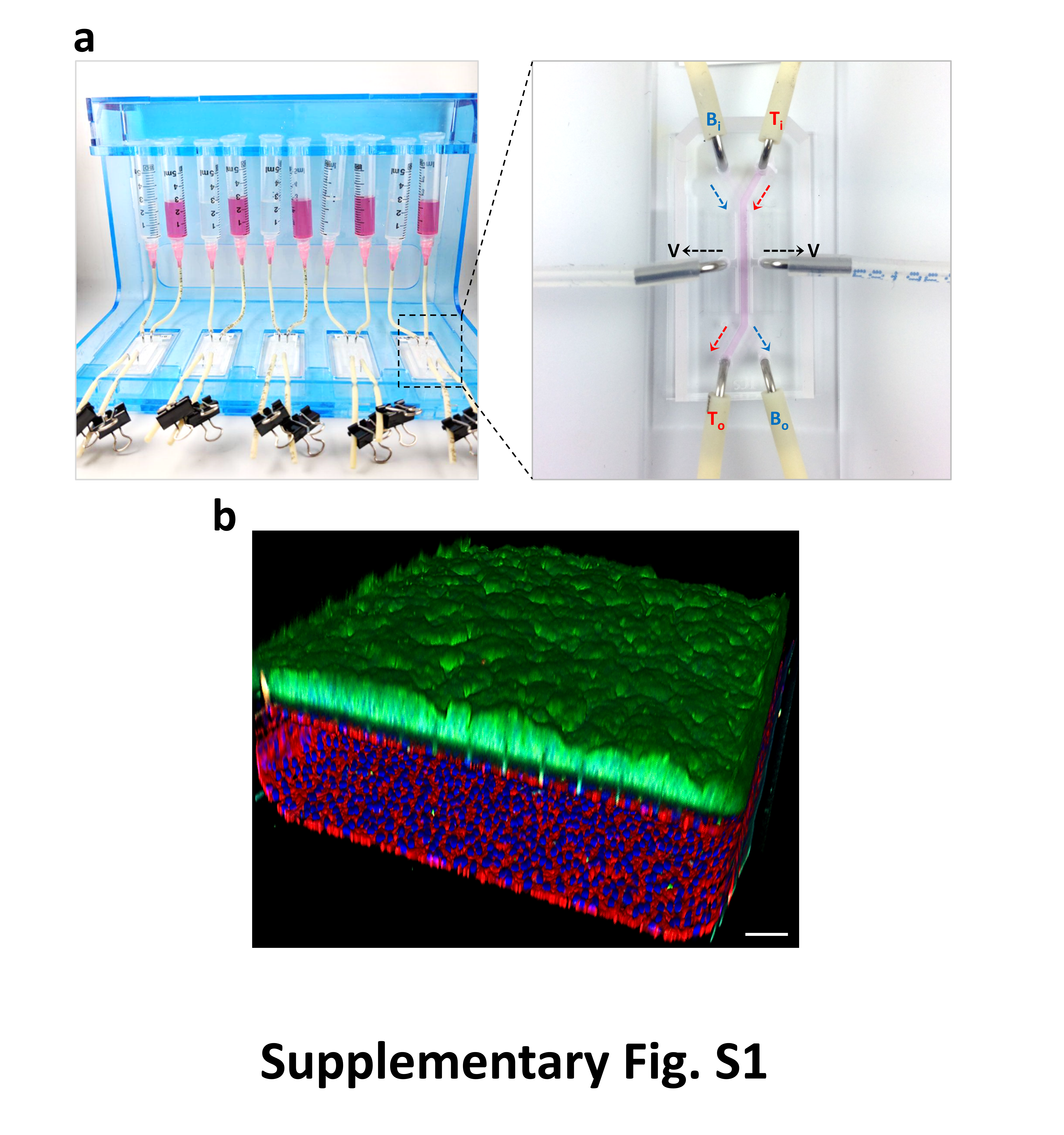

Supplement: Supplementary file 1 — Figure S1 [file 41419_2018_304_MOESM1_ESM.tif]

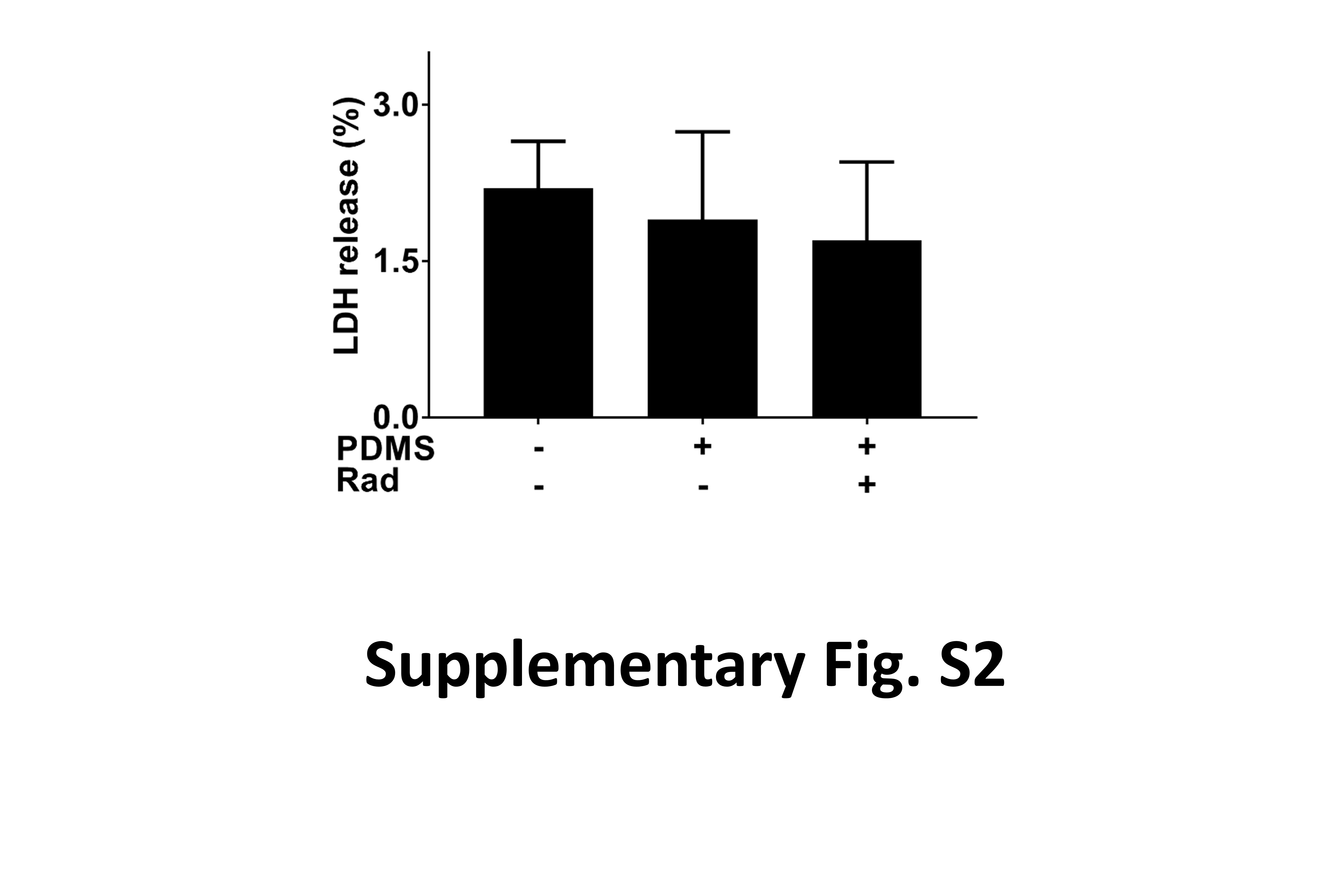

Supplement: Supplementary file 2 — Figure S2 [file 41419_2018_304_MOESM2_ESM.tif]

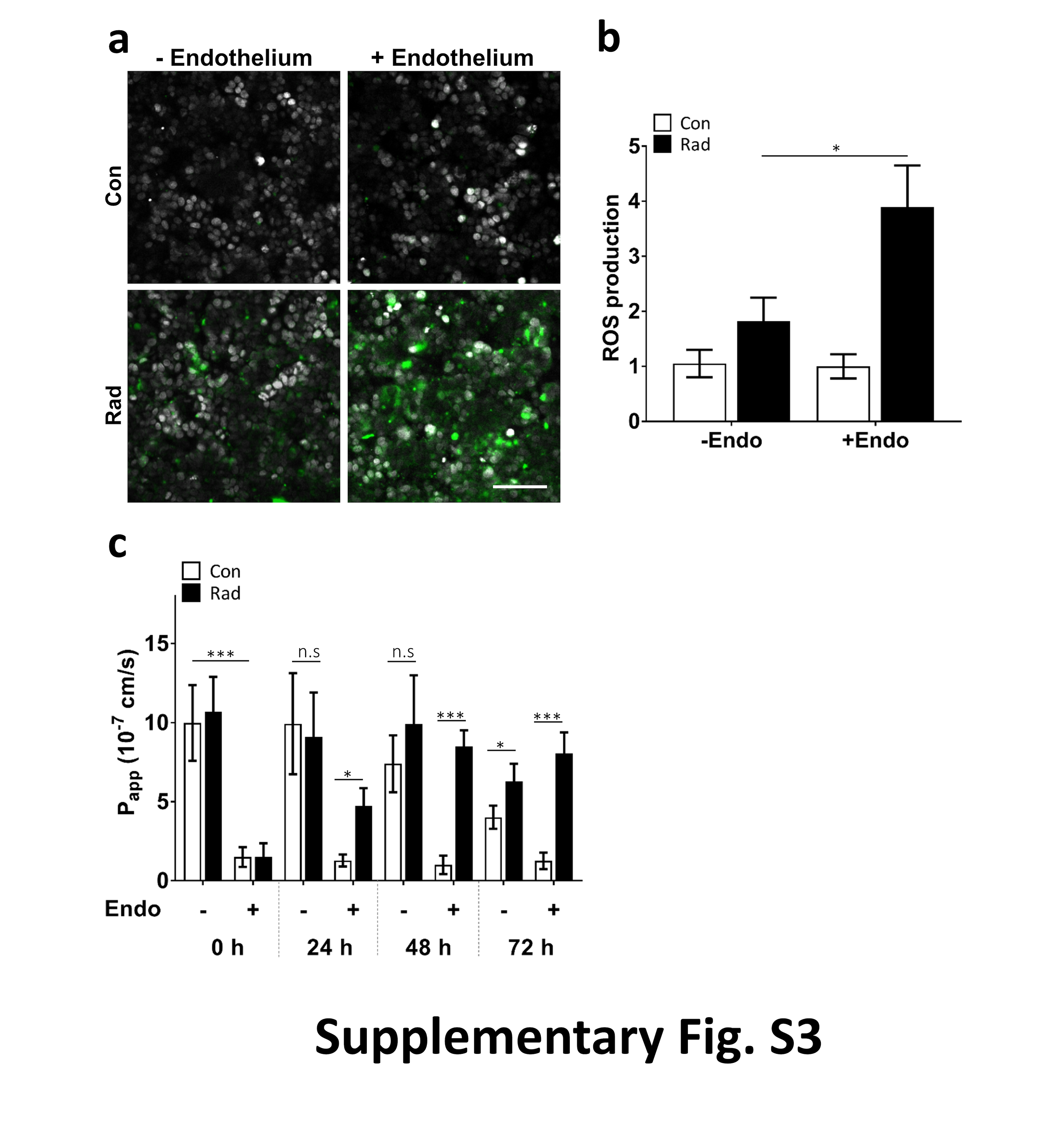

Supplement: Supplementary file 3 — Figure S3 [file 41419_2018_304_MOESM3_ESM.tif]

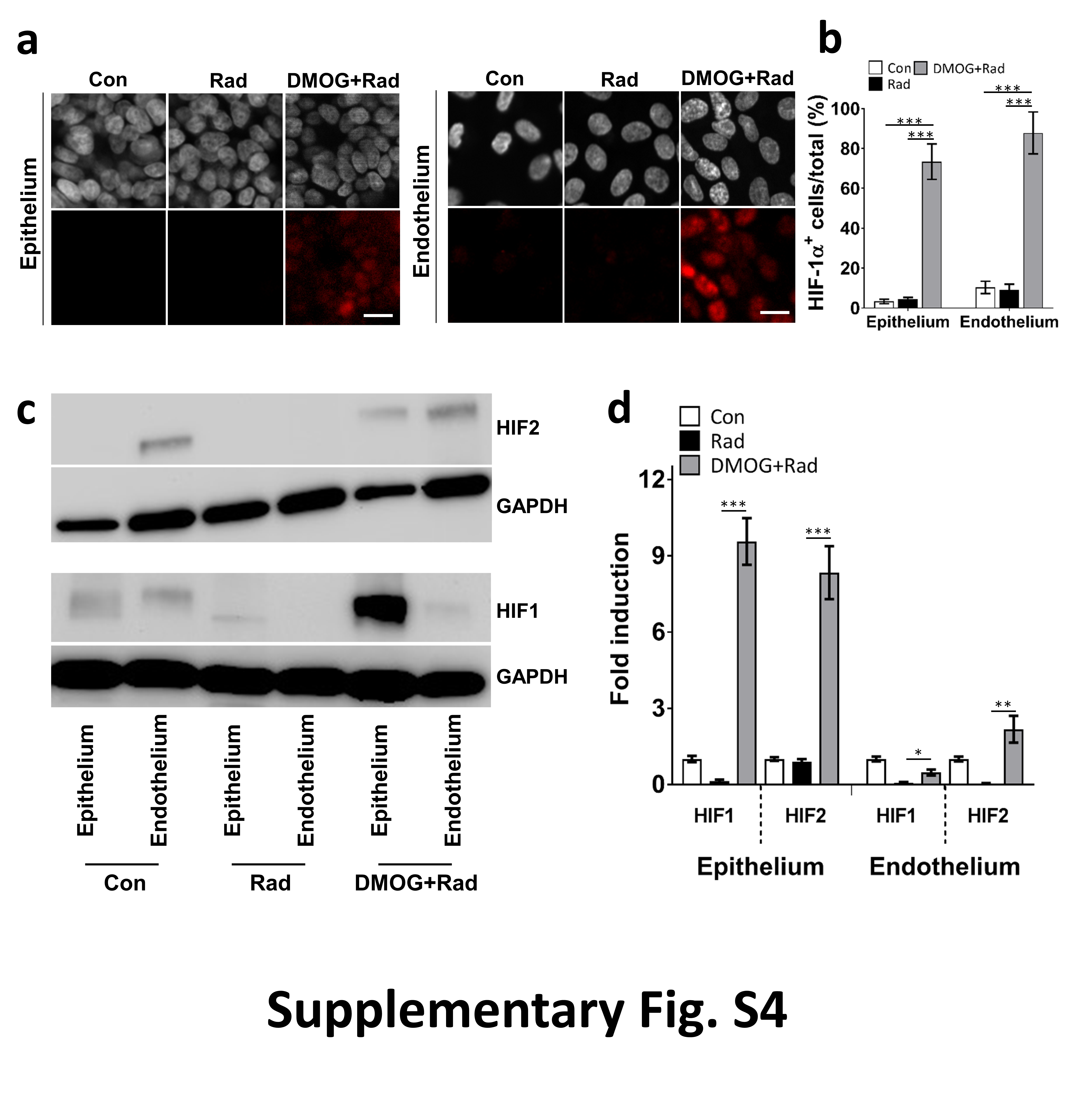

Supplement: Supplementary file 4 — Figure S4 [file 41419_2018_304_MOESM4_ESM.tif]

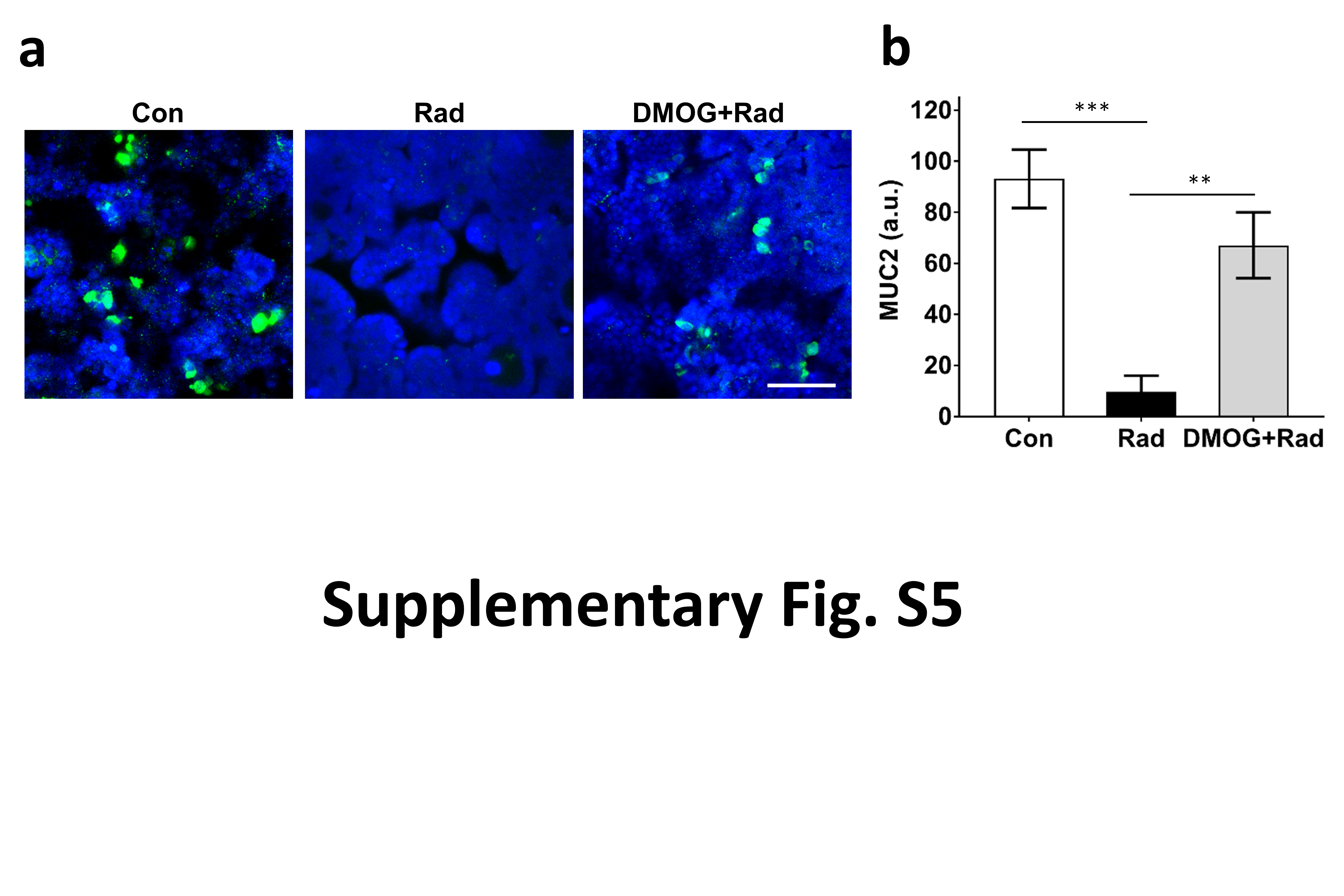

Supplement: Supplementary file 5 — Figure S5 [file 41419_2018_304_MOESM5_ESM.tif]
