# Supplementary material for: Modeling radiation injury-induced cell death and countermeasure drug responses in a human Gut-on-a-Chip
Source: Cell Death Dis. 2018 Feb 14;9(2):223. doi: 10.1038/s41419-018-0304-8 (PMC5833800; doi:10.1038/s41419-018-0304-8)
Supplement: Supplementary file 6 — Supplementary Legend [file 41419_2018_304_MOESM6_ESM.docx]

**Supplementary Figure Legends**

**Supplementary Figure S1.** (**a**) A photographic image of the Gut Chip operating setup (left) running 5 Gut Chip devices composed of clear PDMS elastomer connected to reservoirs containing epithelium (top, red) and endothelium (bottom, clear) media, and a higher magnification photograph of a single device (right). A syringe pump was used to perfuse medium through tubing from top and bottom channels inlets (T_i_ and B_i_, respectively) towards outlet (T_o_ and B_o_, respectively) while mechanical strain exerted by applying suction to the vacuum (V) chambers. (T, top channel; B, bottom channel; I, inlet; o, outlet). (**b**) Representative 3D confocal immunofluorescence micrographic reconstruction of the human villus intestinal epithelium interfaced with a human vascular endothelium surrounding a central lumen that formed inside the Gut Chip (green, villin; VE-Cadherin, red; DAPI-stained nuclei, blue; bar, 100 μm).

**Supplementary Figure S2.** Effect of materials leached from PDMS after exposure to γ-irradiation (8Gy) on the viability of intestinal epithelial cells cultured on the irradiated substrates compared to conventional plastic dishes, as assessed by LDH release.

**Supplementary Figure S3.** (**a**) Radiation-induced changes in intracellular ROS in the intestinal epithelium cultured without (-Endothelium) or with (+Endothelium) endothelium in the lower channel in control (Con) and irradiated (Rad) chips, as visualized using the CellROX Green Reagent (green) (white, DAPI-stained nuclei; bar, 50 μm). (**b**) Quantification of ROS production measured under the conditions described in **a**, expressed as fold change relative to non-irradiated control cells (n=3; ^*^P<0.05). (**c**) Changes in apparent paracellular permeability (*P*_app_) measured by quantfying cascade blue transport across the tissue-tissue interface within the Gut Chip microdevices with (+) and without (-) presence of endothelium in control (Con) and irradiated (Rad) chips 24 h, 48 h and 72 h after exposure to γ-radiation (8Gy) (n=3; ^*^P<0.05, ^***^P<0.001; n.s., not significant).

**Supplementary Figure S4.** Representative images of immunofluorescence staining of nuclei with DAPI (white) at the top and HIF1-α (red) at the bottom in human intestinal epithelial cells and endothelial cells before and after exposure to radiation (8Gy) without (Rad) or with DMOG pre-treatment (DMOG+Rad) (bar, 30 μm). (**b**) Graph showing the quantification of the percentage of epithelial and endothelial cells that expressed HIF1-α (HIF1-α^+^ cells) after exposure to the conditions shown in **a** (n=3; ***P<0.001). (**c**) Western blot analysis of HIF1-α and HIF2-α protein levels in control (Con), irradiated (Rad) and DMOG-treated (DMOG+Rad) chips. (d) Quantification of HIF1-α and HIF2-α levels from Western blots. GAPDH levels are shown to demonstrate equal loading. (Error bars represent mean ± SEM of relative band intensity values for n=4 chips in each group; ^*^P<0.05, ^**^P<0.01, ^***^P<0.001).

**Supplementary Figure S5.** (**a**) Immunofluorescence micrographs of intestinal epithelial cells cultured on-chip under control conditions (Con) versus after exposure to 8Gy radiation without (Rad) or with DMOG pre-treatment (DMOG+Rad), and stained for the intestinal mucin protein, MUC-2 (green) and nuclei (blue, stained with DAPI; bar, 30 μm). (**b**) Quantification of fluorescence intensities (a.u.) of MUC-2 normalized for equal cell numbers under the conditions shown in **d** (n=3; ^**^P<0.01, ^***^P<0.001).
